# Selective breeding for physical inactivity produces cognitive deficits *via* altered hippocampal mitochondrial and synaptic function

**DOI:** 10.3389/fnagi.2023.1147420

**Published:** 2023-04-03

**Authors:** Nathan R. Kerr, Taylor J. Kelty, Xuansong Mao, Thomas E. Childs, David D. Kline, R. Scott Rector, Frank W. Booth

**Affiliations:** ^1^Department of Biomedical Sciences, University of Missouri, Columbia, MO, United States; ^2^Department of Nutrition and Exercise Physiology, University of Missouri, Columbia, MO, United States; ^3^Department of Medical Pharmacology and Physiology, University of Missouri, Columbia, MO, United States; ^4^Dalton Cardiovascular Research Center, University of Missouri, Columbia, MO, United States; ^5^Research Service, Harry S. Truman Memorial Veterans Hospital, University of Missouri, Columbia, MO, United States; ^6^Department of Medicine, Division of Gastroenterology and Hepatology, University of Missouri, Columbia, MO, United States

**Keywords:** hippocampus, physical inactivity, sedentary, neurodegeneration, brain health, physical activity, Alzheimer’s Disease

## Abstract

Physical inactivity is the 4th leading cause of death globally and has been shown to significantly increase the risk for developing Alzheimer’s Disease (AD). Recent work has demonstrated that exercise prior to breeding produces heritable benefits to the brains of offspring, suggesting that the physical activity status of previous generations could play an important role in one’s brain health and their subsequent risk for neurodegenerative diseases. Thus, our study aimed to test the hypothesis that selective breeding for physical inactivity, or for high physical activity, preference produces heritable deficits and enhancements to brain health, respectively. To evaluate this hypothesis, male and female sedentary Low Voluntary Runners (LVR), wild type (WT), and High Voluntary Runner (HVR) rats underwent cognitive behavioral testing, analysis of hippocampal neurogenesis and mitochondrial respiration, and molecular analysis of the dentate gyrus. These analyses revealed that selecting for physical inactivity preference has produced major detriments to cognition, brain mitochondrial respiration, and neurogenesis in female LVR while female HVR display enhancements in brain glucose metabolism and hippocampal size. On the contrary, male LVR and HVR showed very few differences in these parameters relative to WT. Overall, we provide evidence that selective breeding for physical inactivity has a heritable and detrimental effect on brain health and that the female brain appears to be more susceptible to these effects. This emphasizes the importance of remaining physically active as chronic intergenerational physical inactivity likely increases susceptibility to neurodegenerative diseases for both the inactive individual and their offspring.

## 1. Introduction

Physical inactivity is a primary contributor to over 40 chronic diseases and is officially recognized by the World Health Organization as the fourth leading risk factor for mortality, globally (Kohl et al., 2012; Booth et al., 2017; Ruegsegger and Booth, 2017). Among these chronic diseases lies an established link between physical inactivity and neurodegenerative diseases like Alzheimer’s Disease (AD), whereby recent meta-analyses show that physically inactive individuals have a 40% greater risk of developing AD (Kivimäki et al., 2019), while others have shown that those who are physically active have a 32–42% reduction in AD risk (Cunningham et al., 2020). In support, a recent study found that exercise reverses the greatest amount of the gene expression dysregulation associated with AD and may provide the best theoretical (and non-invasive) AD treatment option (Hill and Gammie, 2022). While we know that exercise and physical activity are beneficial to brain health, our understanding of the effects of physical inactivity and sedentary behavior on brain health at the cellular and molecular level remains relatively unexplored.

**Figure 1:**
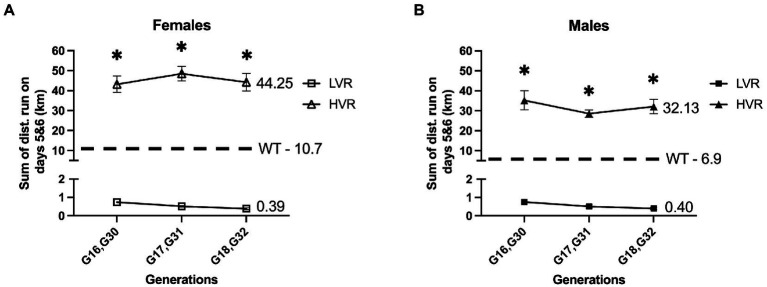
Generational wheel running distances for LVR and HVR relative to representative WT values. **(A)** Running distances on days 5 and 6 for female LVR generations 16–18 (G16-18) and HVR generations 30–32 (G30-32) with female WT Wistar rats serving as a reference point. **(B)** Running distances on days 5 and 6 for male LVR generations 16–18 (G16-18) and HVR generations 30–32 (G30-32) with male WT Wistar rats serving as a reference point. ^*^Denotes significant post-hoc difference relative to LVR following two-way ANOVA.

Our group has attempted to bridge this gap by generating Low Voluntary Runner (LVR) Wistar rats; this is a selective breeding rodent model we have produced by selecting for the primary phenotype of low voluntary running distance when given running wheel access. This selective breeding method has produced rats that run ~20 fold less than their wildtype counterparts when given voluntary running wheel access from 28 to 34 days of age (Figure 1). This has produced a robust model of physical inactivity preference allowing us to study the genetic or epigenetic connection between physical inactivity and brain health (Ruegsegger et al., 2015; Grigsby et al., 2018, 2019, 2020). We have similarly produced High Voluntary Runner (HVR) Wistar rats through selective breeding that display a preference for high levels of physical activity that run ~4 fold more than wildtype rats (Figure 1), allowing us to study both ends of the spectrum as it relates to the intergenerational effect of high and low physical activity selection on brain health and neurodegenerative disease risk.

It is estimated that 35–47% of human physical activity traits and 31% of sedentary behaviors can be explained by genetic factors supporting that physical activity levels are moderately heritable (den Hoed et al., 2013). Building off this, many groups have provided evidence that parental exercise prior to breeding produces beneficial and heritable effects on offspring (Sheldon et al., 2016; Stanford et al., 2017; Mcmillan et al., 2019; Kusuyama et al., 2020). One study in particular found that the offspring of an exercised male rat inherited enhancements in hippocampal neurogenesis, brain mitochondrial citrate synthase activity, and learning and memory (McGreevy et al., 2019). Based on these findings, we hypothesized from an opposing perspective, that selective breeding for a physical inactivity phenotype in our LVR rats also co-selects for heritable effects on the brain that instead act to create deficits in cognition, potentially contributing to a predisposition toward cognitive impairment and subsequent increased neurodegenerative disease risk. We also hypothesize that our HVR will display enhancements in cognition further supporting the previous findings stated above. In this study, we analyzed the innate learning and memory capabilities of male and female LVR and HVR rats under sedentary conditions to determine the heritable contributions that selecting for low or high physical activity levels have produced, along with analyzing the cellular and molecular properties of the dentate gyrus, a region of the brain involved in learning and memory that is heavily affected by neurodegenerative diseases like AD (Rao et al., 2022). Additionally, since women are twice as likely to develop dementia relative to men and experience more severe disease progression (>Alzheimer’s Association, 2020), we further hypothesized that selecting for high and low physical activity will have affected the brains of male and female HVR and LVR in a sex specific manner.

## 2. Materials and methods

### 2.1. Experimental animals

This study utilized male and female LVR, HVR, and wild-type (WT) Wistar rats (4 months of age) that were maintained on a 12:12-h light/dark cycle at 21–22 degrees Celsius. All animals were single housed starting at 8 weeks of age through the remainder of the experiment. Barnes maze behavioral testing began at approximately 14 weeks of age and animals were sacrificed 72 h after Novel Object Recognition (NOR) testing at 16 weeks of age for molecular analysis. A separate set of animals underwent Glucose Tolerance Testing at 16 weeks of age followed by sacrifice for hippocampal mitochondrial analysis 72 h later. An additional set was used for immunohistochemistry of the hippocampus at 16 weeks of age. Water and food were provided *ad libitum* throughout the experiment. In line with the selective breeding design of Koch and Britton (Koch and Britton, 2001), the selective breeding paradigm for LVR and HVR rats consisted of a 13-family line. For each generation, LVR and HVR rats are given free wheel-access from 28 to 34 days of age to verify low and high voluntary wheel-running phenotypes, as performed by us previously (Roberts et al., 2013; Ruegsegger et al., 2015; Grigsby et al., 2018, 2019). Selection is based on nightly running distance and time, which is monitored by Sigma Sport BC 800 bicycle computers, on wheel access nights 5 and 6 (see Figure 1 for recent LVR and HVR generational running data relative to non-breeder founder population). Similar to the breeding scheme of Swallow et al. (1998), LVR consist of the lowest male and lowest female from each family which are chosen as breeders for the next generation while HVR are the opposite, taking the highest male and female for breeding. The experimental rats used in this study were not given running wheel access to avoid any confounding exercise effects on the brain; rather, their litter mates were given free wheel access to confirm the presence of the low and high voluntary running phenotypes. This study utilized generations 17 and 18 (G17 & G18) LVR and generations 31 and 32 (G31 & G32) HVR from multiple families to avoid any litter effects in our results. Ten-week-old male and female WT Wistar rats were purchased from Charles River and were bred in house under identical conditions to LVR and HVR to produce age matched WT rats for experimentation.

### 2.2. Barnes maze testing

To test spatial memory, a gray Barnes maze circular platform (122 cm in diameter) with 20 holes (each being 10.2 cm in diameter) was used similarly to our previous publications (Kelty et al., 2019; Mao et al., 2021). Briefly, the circular platform contained an escape box (28 cm in length, 12.7 cm width, 7.6 cm height) located such that the rat could climb down and in. Each rat was assigned a hole in which the target escape box was located (assigned holes varied among rats to eliminate odor cues when testing). The location of the escape box remained constant for each individual rat throughout the Barnes Maze testing phase. Testing took place during the light cycle between 10:00 AM and 1:00 PM and consisted of two trials a day (1 h apart) for four consecutive days: a familiarization phase (*day 1*), a learning phase (*days 2–4*), and a probe trial testing phase (*day 5*). 24 h later, the animals underwent reversal testing to evaluate their cognitive flexibility. The escape box was placed in a new location (180 degrees from its original position) and the time taken to locate the new position of the escape box and the number of errors made were recorded. Reversal testing was done for 3 consecutive days, 2 trials per day. The Barnes maze was cleaned with 70% ethanol between every animal. ANY-maze software (Stoelting Co., Wood Dale, IL) was used for tracking the rat during testing.

### 2.3. Novel object recognition testing

To evaluate recognition memory, Novel Object Recognition (NOR) testing was performed in a 60 cm length × 60 cm width × 46 cm height arena similar to previous publications (Mao et al., 2021; Kelty et al., 2022). This test is based on the innate preference that rats display toward novel objects rather than objects they are familiar with. This allows for a two-part memory test where rats are familiarized with two objects in part one (familiarization phase), and part two consists of one of the objects being swapped out for a novel object (testing phase). The amount of time spent interacting with both the familiar and novel object is recorded; a rat with intact memory will spend significantly more time interacting with the novel object. ANY-maze tracking software was used to record the time spent exploring both objects. Temperature, sound, and light were controlled throughout the whole experiment, and upon the completion of each testing, 70% ethanol was used to eliminate odor between animals.

### 2.4. Y-maze forced alternation testing

This form of spatial memory testing consists of two trials; a familiarization trial where access to one arm of the maze is blocked, and the rat is placed in the starting arm and given 5 min to explore the starting arm and familiar arm. After an intertrial interval of 1 h, the block to the novel arm is removed and the rat is placed in the starting arm again and given 5 min to explore the entirety of the maze. The amount of time spent in the novel arm of the maze is recorded as an evaluation of the rats’ ability to use their memory abilities to differentiate between the familiar and novel arm.

### 2.5. Glucose tolerance testing

To evaluate systemic glucose handling and insulin sensitivity, a separate set of 4-month-old experimental rats were used for glucose tolerance testing to avoid stress effects on behavioral and molecular outcomes. Rats were fasted for 4 h prior to basal blood glucose readings and continued to be fasted throughout the 2-h duration of blood glucose monitoring. Blood glucose measurements were taken using a Precision Xtra Blood Glucose Monitoring System with Precision Xtra blood glucose strips following tail vein needle puncture. After basal readings, rats received an intraperitoneal injection of glucose (1 g/kg bodyweight) followed by blood glucose measurements 30-, 60-, 90-, and 120-min post glucose injection.

### 2.6. Tissue collection

For molecular analysis, experimental rats underwent euthanasia by CO2 asphyxiation 72 h after all behavioral testing was complete, the brains were rapidly removed and dentate gyrus rich punches [identified using Paxinos and Watson’s rat brain atlas (Paxinos and Watson, 2013)] of 3 mm in diameter were taken from 2 mm thick coronal brain slices obtained using a brain matrix (Braintree Scientific, Braintree, MA). Dentate gyrus punches were flash frozen with liquid nitrogen and stored at −80°C until processing. For immunohistochemistry, rats were anesthetized with isoflurane and perfused with Phosphate Buffered Saline (PBS) followed by 4% paraformaldehyde (PFA) to fix the brain. For hippocampal mitochondrial respiration analysis, rats were anesthetized with isoflurane, decapitated, and the hippocampus rapidly dissected in a cold saline solution.

### 2.7. RNA isolation, cDNA synthesis, and qRT-PCR

Following NOR testing, the dentate gyrus was removed as described above. RNA was isolated from dentate gyrus punches using the Direct-zol RNA Miniprep + DNAse treatment kit (Zymo, R2051). Prior to going into the kit, dentate gyrus punches were placed in TRIzol (Invitrogen, Carlsbad, CA) and homogenized three times for 1 min at 25 Hz using RNase-free, stainless-steel beads and a Tissuelyser (Qiagen, Germantown, MD). To prevent degradation of samples, the samples were set on ice for 1 min between each 1-min homogenization. Next, after going through the kit, RNA was then quantified using a Nanodrop 1,000 (Thermo Scientific, Waltham, MA). For cDNA synthesis, RNA was reverse transcribed using a High-Capacity cDNA Reverse Transcription Kit (Applied Biosystems, Carlsbad, CA). For qRT-PCR, gene-specific primers were constructed using Primer3 (Table 1). cDNA from each sample was assayed in duplicate for the target genes in Table 1 using iTaq Universal SYBR green Supermix (Bio-Rad Laboratories, Hercules, CA). mRNA expression values were quantified by the 2 (−delta delta CT) (Kelty et al., 2019, 2022; Grigsby et al., 2020; Mao et al., 2021) method normalized to *Pgk1*.

**Table 1:** Primers used for qRT-PCR.

Gene	Forward	Reverse	Accession number
*Insr*	CAGTTTGTGGAACGGTGCTG	TGGTAGGGTCATCGGGTTCT	NM_017071
*Irs1*	TTTTCGACACCTCCCTCTGC	CTTGGGTTTGCGCAGGTAAC	NM_012969
*Irs2*	CCTGTGGGTCGGATTTTGGA	GGAAGGCACTGCTGAGTGAT	NM_001168633
*Hk1*	TGGCCTATTACTTCACCGAGC	CGGGAGAGGCCATTCTTCAT	NM_012734
*Pfk*	GCGGAGGAGAGCTAAAACTACA	CCCTGACCGCAGCATTCATA	NM_031715
*Gria1*	GGACAACTCAAGCGTCCAGA	CACAGTAGCCCTCATAGCGG	NM_031608

### 2.8. Western blotting

Following dentate gyrus tissue collection, protein was isolated as previously described (Mao et al., 2021; Kelty et al., 2022). Briefly, the tissue was homogenized in RIPA buffer (50 mM Tris·HCl (pH 8.0), 150 mM NaCl, 1% NP-40, 0.5% sodium deoxycholate, 1% SDS, 1 protease and phosphatase inhibitor cocktail) using stainless steel beads and a Tissuelyser (Qiagen). Homogenized samples then underwent centrifugation at 12,000 *g* for 10 min with the supernatant protein concentrations obtained using the BCA assay (Pierce Biotechnology, Rockford, IL). 22.5 micrograms of protein in loading buffer were loaded onto 4–15% SDS-PAGE gels. Proteins were then transferred to PVDF membranes, and all blots were incubated with NoStain Protein Labeling Reagent (Thermofisher) for total protein normalization and to verify equal loading in each lane. After, Blocker FL 10x Fluorescent Blocking Buffer (Thermofisher) was used to block nonspecific binding. Primary antibodies (rabbit and mouse polyclonal) AMPA receptor 1 (GluA1) (1:1000, Cell Signaling D4N9V, 100 kd), Amyloid Precursor Protein (APP) (1:1000, Cell Signaling E4H1U, 100–140 kd), Tau (1:1000, Cell Signaling Tau46, 50–80 kd), phospho-Tau (pTau Thr181) (1:1000, Cell Signaling D9F4G, 50–80 kd) diluted in Tris-buffered saline + Tween20 (TBS-T) with 5% BSA were applied overnight at 4°C. Anti-Rabbit Alexa Fluor 633 or Anti-Mouse Alexa Fluor 800 secondary antibodies were used at a dilution of 1:3000 (Thermofisher) and applied for 1 h at room temperature. The Precision Plus Protein Dual Color Standards ladder (BioRad) was used to verify molecular weight. Imaging was done using the iBright FL1500 and rolling background band volume normalized to total protein was quantified using the iBright analysis software (Thermofisher).

### 2.9. Immunohistochemistry

Rats underwent cardiac perfusion with 4% PFA and brains were extracted and stored in 4% PFA overnight. All brains underwent a sucrose gradient (3 days in 20% sucrose followed by 3 days in 30% sucrose) and then brains were placed in OCT and sliced 40 microns thick throughout the dorsal portion of the hippocampus. Slices were then stored in a cryoprotectant solution until time of staining. For staining, 6 slices were used per animal (every third slice in sequential order were chosen) with one slice used as a negative control with no primary antibody. Slices were blocked for 30 min [10% Normal Donkey Serum (NDS)] in 0.3% Triton-PBS followed by overnight incubation with primary antibodies anti-rabbit Doublecortin (DCX) (1:500, Cell Signaling 4604S) and anti-mouse NeuN (1:500, Millipore Sigma MAB377) in 1% NDS and 0.3% Triton-PBS. The following day consisted of a 2-h incubation in 0.3% Triton-PBS, containing 1% NDS with Anti-Rabbit *CF* 488A (1:200, Millipore Sigma A21206) and Anti-Mouse *CF* 555 (1:500, Millipore Sigma A31570) secondary antibodies. Slices were then mounted onto gelatin coated slides, coverslipped with ProLong Gold, and sealed with nail polish (Lima-Silveira et al., 2022; Martinez et al., 2022). Images were acquired using an upright fluorescence microscope (BX51, Olympus, Japan) equipped with an ORCA-ER CCD camera (Hamamatsu Photonics) at 10× magnification. Image analysis was done using ImageJ.

### 2.10. Mitochondrial respiration analysis *via* Oroboros O2k

To evaluate mitochondrial function in the hippocampus of LVR, isolated hippocampi were placed in ice cold ATP buffer at time of collection then transferred to ice cold mitochondrial isolation buffer for mincing. Tissue was further homogenized using a Teflon homogenizer at slow speeds. Next, homogenate was transferred to a 15 mL conical vial and centrifuged at 1,500 × *g* for 5 min. Supernatant was then transferred to a percoll gradient (40, 23, and 15% percoll) and centrifuged at 9,600 × *g* for 13 min. This produces two rings of separation, with the bottom ring being mitochondria. The mitochondrial ring was extracted and pelleted following another centrifugation at 8,000 × *g* for 10 min. The resulting mitochondrial pellet is resuspended in 150 μL of mitochondrial phosphate buffer (MiPO3). Mitochondria were given 30 min on ice prior to being loaded in the Oroboros for assessment of mitochondrial respiration using high-resolution respirometry (Oroboros Oxygraph-2 k, Oroboros Instruments, Innsbruck, Austria) (Cunningham et al., 2021, 2022; Queathem et al., 2022). The mitochondrial respiration protocol following addition of mitochondria to the Oroboros consisted of malate (2 mM) and glutamate (5 mM) for state 2 respiration (measuring complex I leak with substrate rate limiting), ADP (1,000 μM) for state 3 – Complex I (measuring oxidative phosphorylation through complex I), succinate (10 mM) for state 3 – complex I + II (measuring oxidative phosphorylation capacity through both complex I and II), FCCP (0.25 μM) for uncoupled (maximal oxygen consumption), and Cyto C (5 μM) to verify quality of mitochondrial preparations. The Oroboros DatLab system was used for analysis. Isolated mitochondria for each sample underwent protein quantification *via* BCA assay (Pierce Biotechnology) for normalization of mitochondrial loading.

### 2.11. Electrophysiology and LTP recordings

4–6-week-old female WT and LVR were anesthetized, decapitated, and the brain rapidly removed and placed in oxygenated ice cold high magnesium-low calcium artificial cerebrospinal fluid (ACSF) (124 mM *NaCl*, 2 mM *KCl*, 1.25 mM *KH_2_PO_4_*, 3.5 mM *MgSO_4_*, 0.5 mM *CaCl_2_*, 26 mM *NaHCO_3_*, and 10 mM D-Glucose). Brains were then cut into 400 μm horizontal sections using a vibratome while submerged in oxygenated ice-cold high magnesium-low calcium ACSF. Slices were then transferred to 32°C recording ACSF (124 mM *NaCl*, 2 mM *KCl*, 1.25 mM *KH_2_PO_4_*, 2 mM *MgSO_4_*, 26 mM *NaHCO_3_*, 2 mM *CaCl_2_*, and 10 mM D-Glucose) and left to rest for 1 h. A slice would then be transferred to the recording chamber and given ~30 min to equilibrate. Next, a bipolar stimulating electrode was positioned in the stratum radiatum layer of CA3, and the recording pipette (filled with recording ACSF) was positioned in the stratum radiatum region of the CA1. An input output curve was then generated, and stimulation strength was set to 40% of maximum slope. Baseline recordings were made for the next 20 min after which LTP was induced by tetanic stimulus of 100 Hz (4 trains of 100 Hz, 20 s train interval) and slices were recorded for an additional 40 min. fEPSP slopes were analyzed using Clampfit 10 software (Molecular Devices).

### 2.12. Statistical analysis

Statistical analysis was performed in GraphPad Prism. For Barnes maze acquisition and reversal testing, a Repeated Measures (RM) two-way ANOVA was used to make group (WT, LVR, and HVR) versus trial comparisons, and if main effects or interactions were present, a Tukey *post-hoc* test was used to detect any further differences. A one-way ANOVA was used for the Barnes maze probe trial. For NOR testing, a student’s unpaired *t*-test was used to detect within group differences between the training and testing phase while a one-way ANOVA was used to test for group differences in the testing phase, followed by Tukey *post-hoc* analysis. A one-way ANOVA was used for all molecular comparisons consisting of three groups (WT, LVR, and HVR) followed by Tukey *post-hoc* analysis. For mitochondrial respiration, LTP, and immunohistochemistry analysis, a student’s unpaired t-test was used for comparisons between WT and LVR. A RM 2-way ANOVA was also used for the glucose tolerance testing followed by Sidak *post-hoc* analysis. *P* values <0.05 were considered significant and data is presented as mean + SEM.

## 3. Results

### 3.1. Female LVR display deficits in spatial learning and memory while male LVR do not

**Figure 2:**
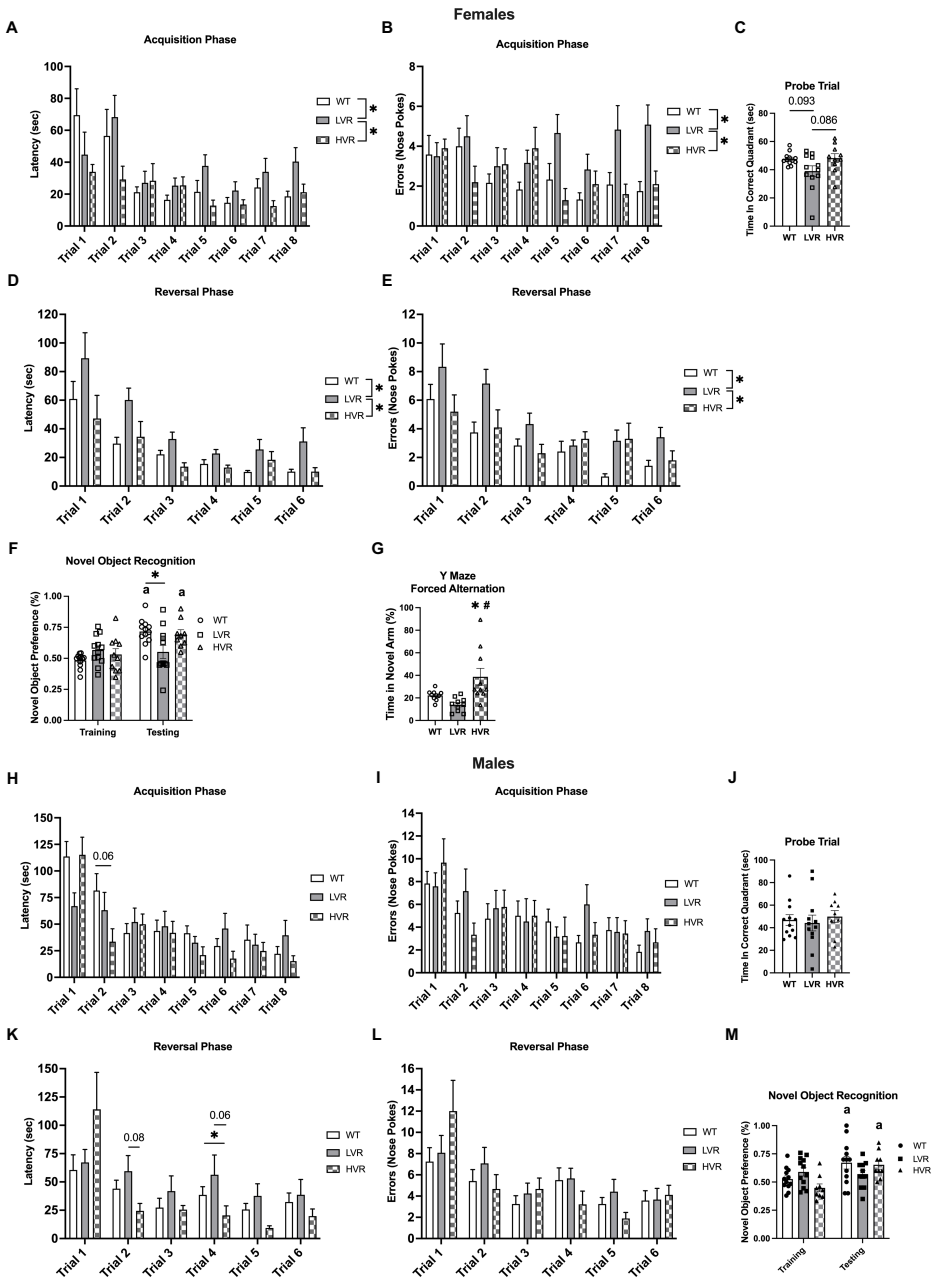
Female LVR, but not male LVR, display deficits in spatial learning and memory. Barnes maze acquisition phase **(A,B)**, probe trial **(C)**, and reversal phase **(D,E)** results between female WT (white bars, *N* = 12), LVR (gray bars, *N* = 12), and HVR (checkered bars, *N* = 10). Novel Object Recognition results for female LVR, WT, and HVR **(F)**. Y-Maze Forced Alternation results for female LVR, WT, and HVR **(G)**. Barnes maze acquisition phase **(H,I)**, probe trial **(J)**, and reversal phase **(K,L)** results between male WT (white bars, *N* = 12), LVR (gray bars, *N* = 12), and HVR (checkered bars, *N* = 9). Novel Object Recognition results for male LVR, WT, and HVR **(M)**. ^*^Denotes main effect of group in two-way RM ANOVA or post-hoc difference. ^a^ Denotes within group difference between training and testing phase. ^#^Denotes *post-hoc* difference relative to LVR. Values expressed as mean + SEM.

To evaluate how selecting for high and low voluntary running phenotypes has affected learning and memory, LVR and HVR underwent Barnes maze and Novel Object Recognition (NOR) testing with age matched WT rats serving as a reference group. For Barnes Maze testing in female rats, LVR took significantly longer to locate the escape box relative to both WT and HVR during the acquisition phase {main effect for group [*F*(2, 31) = 3.430; *p* = 0.045] and trial [*F*(3.739, 115.9) = 7.741; *p* = 0.0001]}, with a trending interaction [*F*(14, 217) = 1.635; *p* = 0.072] (Figure 2A). Along with LVR taking significantly longer to locate the escape box, they also committed more errors while finding the escape box across the acquisition trials relative to both WT and HVR {group main effect [*F*(2, 31) = 9.178; *p* < 0.001]} and a trending interaction [*F*(14, 217) = 1.616; *p* = 0.077] (Figure 2B). The acquisition phase was followed by a probe trial which showed a trend for LVR to spend less time in the correct quadrant relative to WT and HVR, but this difference did not reach statistical significance {trending group main effect [*F*(2, 31) = 3.237; *p* = 0.053] with Tukey post-hoc analysis revealing trending group differences: LVR vs. WT *p* = 0.093; LVR vs. HVR *p* = 0.086} (Figure 2C). Next, groups began the reversal testing phase where again, LVR took significantly longer to locate the escape box at its new location across trials relative to WT and HVR {group [*F*(2, 31) = 5.841; *p* = 0.007] and trial main effect [*F*(1.896, 58.79) = 23.15; *p* < 0.0001]} (Figure 2D). The errors committed across trials closely matched the latency data by also showing LVR commit significantly more errors across trials relative to both WT and HVR {group [*F*(2, 31) = 7.588; *p* = 0.002] and trial main effect [*F*(3.088, 95.71) = 13.26; *p* < 0.0001]} (Figure 2E). NOR testing, a simple test designed to evaluate recognition memory, in female LVR, WT, and HVR supported the Barnes Maze testing findings as female WT and HVR rats spent significantly more time interacting with the novel object during the testing phase relative to the training phase, while LVR did not. A one-way ANOVA was used to detect group differences in the testing phase and found that LVR spent significantly less time interacting with the novel object compared to WT [*F*(2, 30) = 4.891; *p* = 0.0145] (Figure 2F). Y maze forced alternation testing was also done on the female experimental groups and demonstrated that HVR spent significantly more time in the novel arm relative to WT and LVR, providing evidence that female HVR have enhancements in spatial memory relative to WT {main effect [*F*(2, 27) = 7.741; *p* = 0.002] for time spent in the novel arm} (Figure 2G).

Male WT, LVR, and HVR underwent behavioral testing identical to that of the females. For the acquisition phase of the Barnes maze, no post-hoc differences were noted for LVR in regard to latency, while HVR had a trending decrease in latency to locate the escape box for trial 2 relative to WT (*p* = 0.06) {significant interaction [*F*(14, 210) = 1.799; *p* = 0.04] and main effect for trial [*F*(4.394, 131.8) = 13.39; *p* < 0.0001]} (Figure 2H). There were no group differences noted for errors made when locating the escape box, only a main effect for trials [*F*(5.316, 159.5) = 5.299; *p* < 0.0001] indicating improvement across trials (Figure 2I). There were no group differences noted for the probe trial following the acquisition phase either (Figure 2J). For the reversal phase of the Barnes maze, a few post-hoc differences were noted with LVR trending to take longer to locate the escape during trial 2 relative to WT (*p* = 0.08) and HVR taking significantly less time to locate the escape box during trial 4 relative to WT and LVR (*p* = 0.06) {significant interaction [*F*(10, 150) = 2.402; *p* = 0.011] and main effect for trial [*F*(2.483, 74.48) = 10.93; *p* < 0.0001]} (Figure 2K). Again, we saw no group differences in the number of errors made across trials during the reversal phase, only a main effect for trials [*F*(3.212, 96.36) = 9.399; *p* < 0.0001] (Figure 2L). Despite the lack of differences seen in the Barnes maze testing for the males, NOR testing results were similar to that seen in females. Male WT and HVR spent significantly more time with the novel object during the testing phase relative to the training phase, while male LVR did not. However, a one-way ANOVA did not detect any group differences in the testing phase [*F*(2, 29) = 1.575; *p* = 0.2242] (Figure 2M).

### 3.2. Female LVR display reductions in hippocampal mitochondrial respiration, LTP induction, and neurogenesis while HVR have increased hippocampal volumes

**Figure 3:**
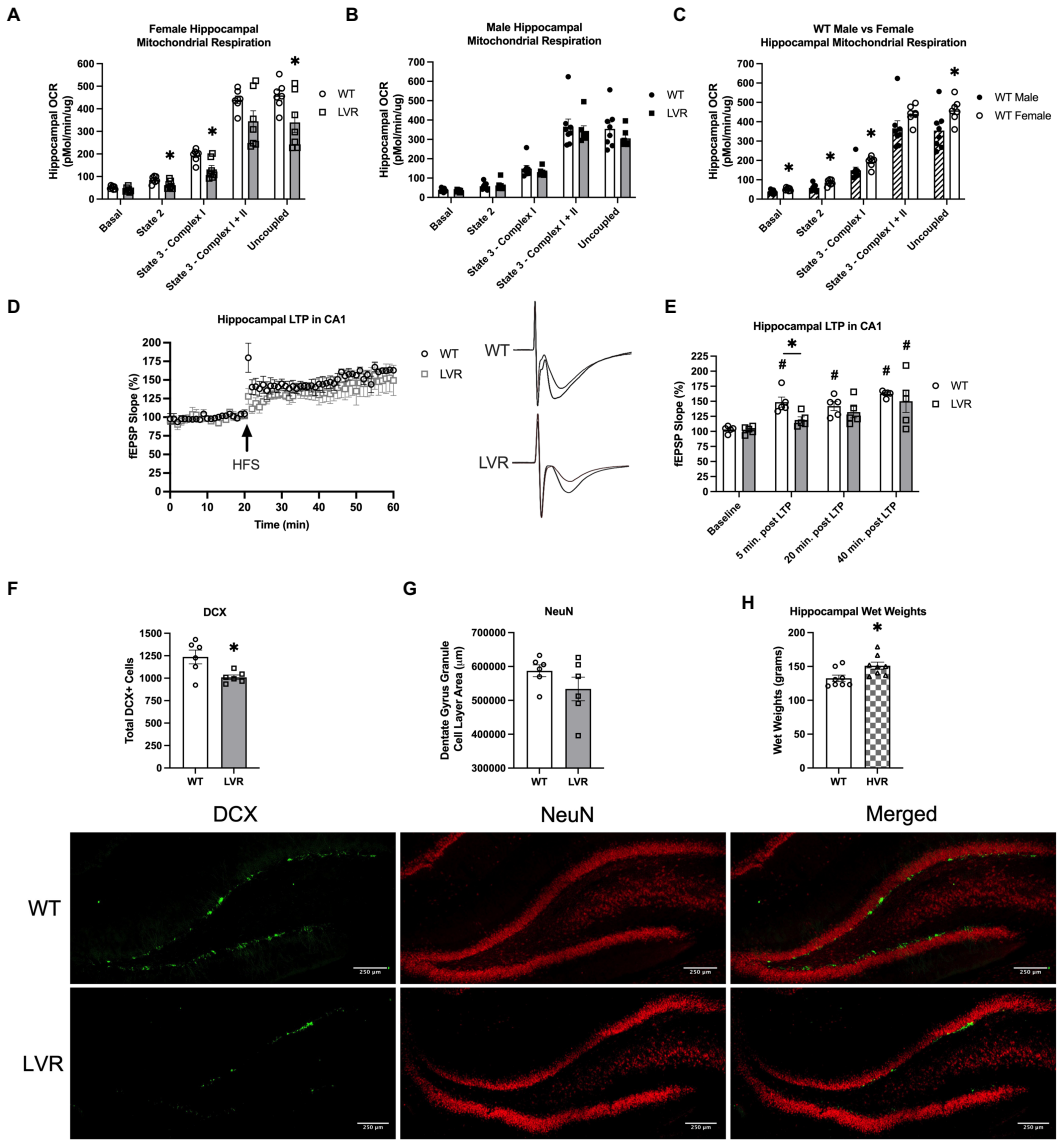
Female LVR have deficits in hippocampal mitochondrial respiration, LTP induction, and neurogenesis while HVR have greater hippocampal volume. Oroboros mitochondrial respiration analysis of the hippocampus between **(A)** female WT (white bars, *N* = 7) and LVR (gray bars, *N* = 7), **(B)** male WT (*N* = 8) and LVR (*N* = 7), and **(C)** male (hashed bars) and female WT. **(D)** LTP recordings in the CA1 region of the hippocampus in female WT (*N* = 5) and LVR (*N* = 5) with representative traces. **(E)** Statistical analysis of LTP recordings between female WT and LVR. **(F)** Number of DCX positive cells in the dentate gyrus of WT (*N* = 6, 6 slices per N) and LVR (*N* = 6, 6 slices per N). **(G)** Total area of NeuN staining in the granule cell layer of the dentate gyrus in WT (*N* = 6, 6 slices per N) and LVR (*N* = 6, 6 slices per N). Representative images of DCX and NeuN staining of the dentate gyrus in female WT and LVR underneath figures (scale = 250 μm). **(H)** Hippocampal wet weights in female WT (*N* = 8) and HVR (*N* = 8). ^*^Denotes significant difference between groups based on Student’s *t*-test. ^#^Denotes significant *post-hoc* difference relative to within group baseline. Values expressed as mean + SEM.

Based on our behavioral testing results demonstrating learning and memory impairments in female LVR rats, we next evaluated mitochondrial function in the hippocampus of these rats, as impairments in brain mitochondrial function are a key sign of AD (Swerdlow, 2018; Perez Ortiz and Swerdlow, 2019; Sharma et al., 2021). Compared to female WT rats, LVR’s had significant reductions in State 2 (*p* = 0.023), State 3 – Complex I (*p* = 0.009), and Uncoupled respiration (*p* = 0.037) (Figure 3A). In contrast, there were no significant differences in oxygen consumption between male LVR and WT rats (Figure 3B). However, female WT had higher levels of hippocampal mitochondrial respiration compared to male WT, including males having significant reductions in basal mitochondrial respiration (*p* = 0.02), State 2 (*p* = 0.007), State 3 – Complex I (*p* = 0.044), and uncoupled respiration (*p* = 0.032) (Figure 3C). These findings support that selecting for physical inactivity coincided with major deficits in hippocampal function, specifically in female LVR, that likely promotes neurodegeneration and AD risk, while male LVR rats appear to be protected from these effects. Basal differences in hippocampal mitochondrial bioenergetics between males and females is a novel finding that may provide some explanation for why females are at greater risk for AD and experience more rapid disease progression relative to males.

Another key connection between physical activity and neurodegenerative disease risk is that physical activity has been demonstrated to enhance adult neurogenesis and long-term potentiation (LTP) in the hippocampus, both of which play a neuroprotective role and are believed to be necessary for learning and memory to take place (van Praag et al., 1999a,b; Sahay et al., 2011; Moreno-Jiménez et al., 2019). Therefore, we sought to address whether selecting for physical inactivity had an opposite effect, acting to blunt neurogenesis and long-term potentiation. Our data thus far has suggested that selecting for low physical activity has produced the most significant heritable cognitive deficits in female LVR. Based on this, we performed electrophysiological LTP recordings in the CA1 region of the hippocampus in female LVR and WT rats (Figure 3D). In LVR rats, a one-way ANOVA revealed that field extracellular post-synaptic potential (fEPSP) slope did not significantly increase from baseline until 40 min. Post LTP induction [*F*(3, 16) = 3.506; *p* = 0.0398], while in WT we saw an immediate significant increase in fEPSP slope following LTP induction that lasted the full 40 min. [*F*(3, 16); *p* < 0.0001] (Figure 3E). Additionally, fEPSP slope was significantly less in LVR relative to WT at 5 min. Post LTP induction (*p* = 0.017) (Figure 3E). Next, female LVR and WT hippocampal slices were stained for DCX to determine the number of immature neurons and NeuN for the number of mature neurons in the dentate gyrus region of the hippocampus to evaluate basal rates of adult neurogenesis. DCX and NeuN staining revealed that female LVR have significantly less DCX+ cells compared to WT (*p* = 0.019) (Figure 3F), but no differences were detected in the amount of NeuN staining area in the dentate gyrus granule cell layer (Figure 3G). This suggests that the basal rate of neurogenesis is likely reduced in female LVR as there are fewer immature neurons present in the dentate gyrus. Along with mitochondrial dysfunction, reductions in neurogenesis and LTP are both key signs of AD (Rodríguez et al., 2008; Rodríguez and Verkhratsky, 2011) supporting that female LVR display a cognitively impaired phenotype. While we primarily focused on female LVR and WT in this experiment, we have also collected data showing that female HVR have significantly greater hippocampal wet weights compared to WT (Figure 3H). A higher cognitive reserve is believed to result in a resiliency against AD pathology (Stern, 2006; Barnes, 2015), suggesting that selecting for high physical activity preference has produced a heritable enhancement on brain reserve volume.

### 3.3. Female LVR have deficits in AMPA receptor expression but no changes in amyloid precursor protein or tau phosphorylation

**Figure 4:**
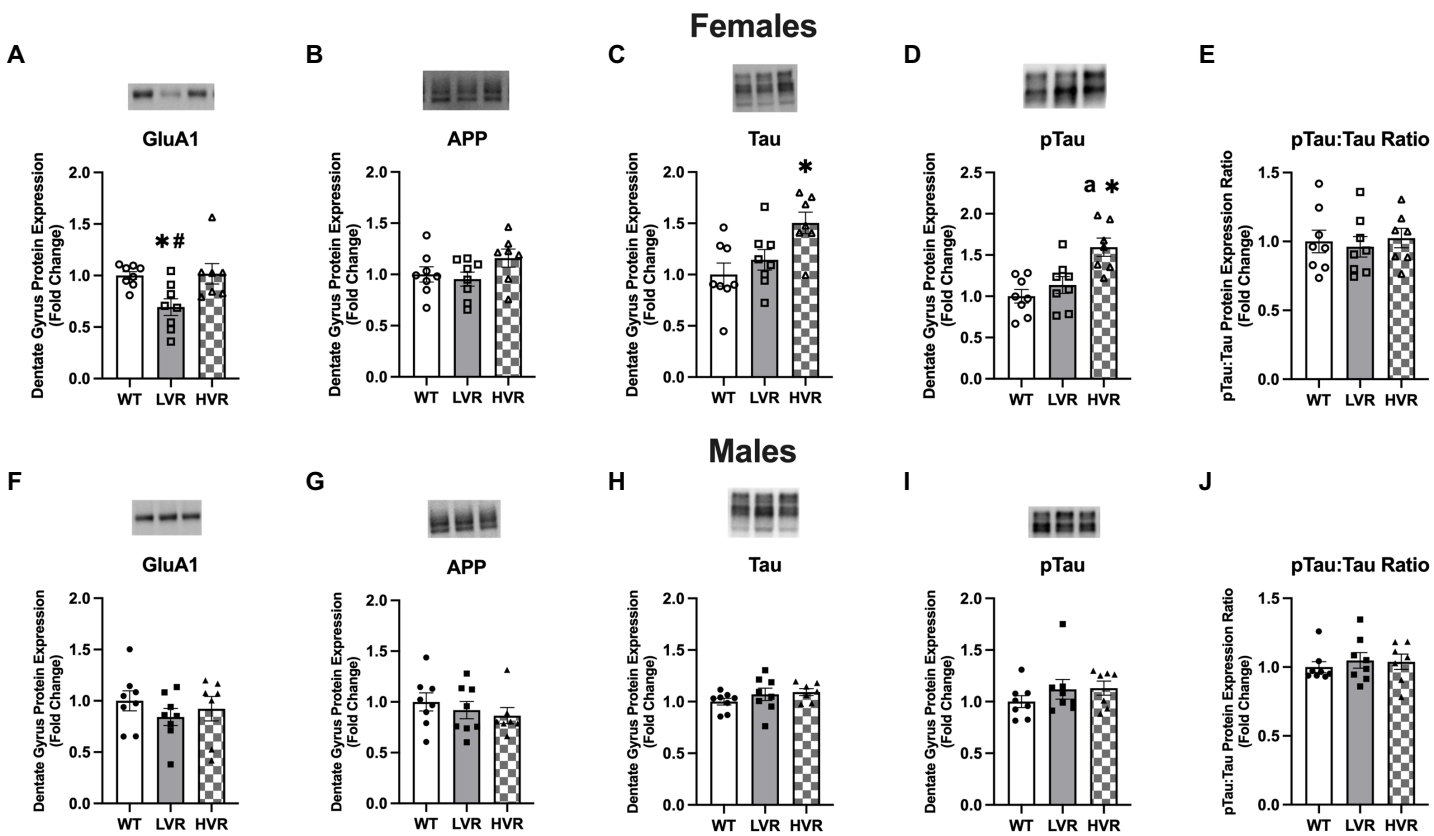
Female LVR have reductions in dentate gyrus GluA1 protein expression. **(A–E)** Protein expression levels of GluA1, APP, Tau, pTau, and pTau:Tau Ratio in female WT (*N* = 8), LVR (*N* = 8) and HVR (*N* = 7). **(F–J)** Protein expression levels of GluA1, APP, Tau, pTau, and pTau:Tau Ratio in male WT (*N* = 8), LVR (*N* = 8) and HVR (*N* = 7). ^a^The significant difference between LVR and HVR. ^*^Denotes significant Tukey *post-hoc* difference relative to WT following one-way ANOVA. ^#^Denotes significant Tukey *post-hoc* difference relative to HVR following one-way ANOVA. Values expressed as mean + SEM.

To evaluate molecular changes in the hippocampus associated with the selection of high and low physical activity, we analyzed the expression of AD-related proteins in the dentate gyrus region of the brain in male and female LVR, WT, and HVR. One important protein that has recently been suggested to be an initiator in AD pathogenesis is the AMPA receptor subunit GluA1 (Guntupalli et al., 2016; Qu et al., 2021). In females, western blot analysis revealed reductions in GluA1 protein levels in LVR compared to WT and HVR {main effect [*F*(2, 20) = 5.985; *p* = 0.009]} (Figure 4A). Next, we analyzed the protein levels of APP, a precursor to the amyloid beta protein, and Tau, which becomes hyperphosphorylated and accumulates in the brain during AD. There were no differences in APP protein expression between groups (Figure 4B). Surprisingly, Tau and pTau protein expression was higher in HVR relative to both WT and LVR {Tau main effect [*F*(2, 20) = 5.754; *p* = 0.011] and pTau main effect [*F*(2, 20) = 9.726; *p* = 0.001]} (Figures 4CD). However, there were no differences in the ratio of pTau to total Tau between groups (Figure 4E), leading us to hypothesize these increases in Tau seen in HVR are physiological (greater axonal and dendritic branching) rather than pathological. The lack of differences in APP, Tau, and pTau levels between LVR and WT is not overly surprising as the rats used in these studies are relatively young (4 months old) adults so accumulation of these proteins may not have occurred yet. Interestingly, there were no group differences seen in male LVR, WT, and HVR (Figures 4F–J). This supports our behavioral testing findings that male LVR do not appear to have cognitive deficits to the same extent as female LVR.

### 3.4. Female HVR display enhancements in brain glucose metabolism and hippocampal volume

**Figure 5:**
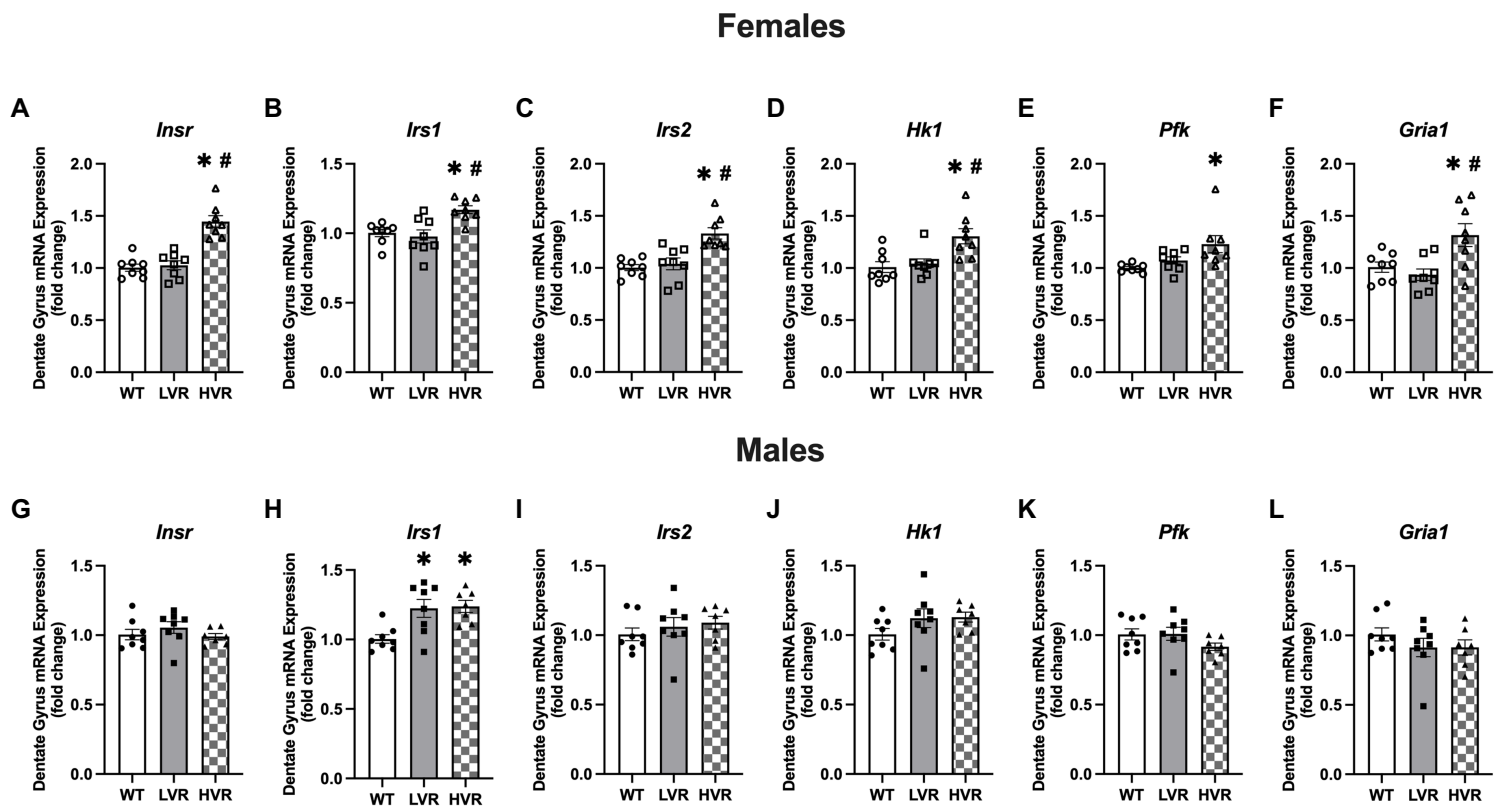
Female HVR have enhancements in brain insulin signaling and glucose metabolism. **(A–F)** Transcript expression levels of *Insr*, *Irs1*, *Irs2*, *Hk1*, *Pfk*, and *Gria1* in female WT (*N* = 8), LVR (*N* = 8), and HVR (*N* = 7). **(G–L)** Transcript expression levels of *Insr*, *Irs1*, *Irs2*, *Hk1*, *Pfk*, and *Gria1* in male WT (*N* = 8), LVR (*N* = 8), and HVR (*N* = 7). ^*^Denotes significant Tukey *post-hoc* difference relative to WT following one-way ANOVA. ^#^Denotes significant Tukey *post-hoc* difference relative to LVR following one-way ANOVA. Values expressed as mean + SEM.

Recent work has highlighted the importance of brain insulin sensitivity in AD (de La Monte, 2012; Kerr and Booth, 2022); insulin signaling in the brain has been demonstrated to inhibit neuronal apoptosis as well as regulate tau phosphorylation and amyloid beta clearance (Neumann et al., 2008; Kellar and Craft, 2020). Similarly, another key sign of AD is brain glucose hypometabolism which is believed to produce mitochondrial deficits which have been shown to lead to altered APP processing and increase amyloid-beta and tau phosphorylation (Mosconi, 2005; Dewanjee et al., 2022). Therefore, we analyzed the gene expression levels of the insulin receptor pathway (Insulin receptor, *Insr*, Insulin receptor substrate 1, *Irs1*, and Insulin receptor substrate 2, *Irs2*) and the rate limiting enzymes in glycolysis (Hexokinase, *Hk1*, and Phosphofructokinase, *Pfk*) in the dentate gyrus. To further determine potential genotype differences in hippocampal glutamate signaling (a potential molecular mechanism of AD) we measured the GluA1 AMPA receptor subunit at the transcript level, *Gria1*, across LVR, WT, and HVR. Regarding insulin receptor signaling in the females, HVR showed greater transcript levels of *Insr*, [*F*(2, 21) = 29.20; *p* < 0.0001], *Irs1*, [*F*(2, 21) = 7.985; *p* = 0.003], and *Irs2*, [*F*(2, 21) = 12.38; *p* = 0.0003] relative to both WT and LVR (Figures 5A–C), suggesting an altered insulin signaling mechanism in response to selection of high physical activity.

For the rate limiting enzymes in glycolysis, HVR also had significantly greater *Hk1* expression relative to both WT and HVR [*F*(2, 21) = 8.041; *p* = 0.003] and *Pfk* expression relative to WT [*F*(2, 21) = 5.967; *p* = 0.009] (Figures 5D,E). *Gria1* expression was also significantly higher in HVR relative to WT and LVR [*F*(2, 21) = 6.354; *p* = 0.007] (Figure 5F). Altogether, this demonstrates that female HVR may have enhanced insulin signaling and glucose metabolism in the dentate gyrus, both of which are believed to play a neuroprotective role in the context of AD (Morris and Burns, 2012; Duran-Aniotz and Hetz, 2016). Moreover, this may provide evidence of a potential molecular mechanism for how physical activity reduces the risk of AD. The *Gria1* data coupled with its expression at the protein level suggest that our selective breeding has produced opposing effects on its expression profiles, further emphasizing its importance as a link between physical activity levels and AD risk.

In contrast to the above findings in females, there were few differences in insulin signaling, glycolysis, and AMPA receptor expression levels across male WT, LVR, and HVR rats. The only significant difference is in *Irs1* expression levels which unexpectedly showed that *Irs1* transcript levels were significantly higher in both LVR and HVR relative to WT [*F*(2, 20) = 7.673; *p* = 0.003] (Figure 5H). This provides another example of the stark difference between male and female LVR, where male LVR rats again appear to be spared from any major deficits in the brain and may actually display some compensatory protection against brain insulin resistance in contrast to their female counterparts.

### 3.5. Male LVR display signs of systemic insulin resistance and obesity, while female LVR are protected from these effects

**Figure 6:**
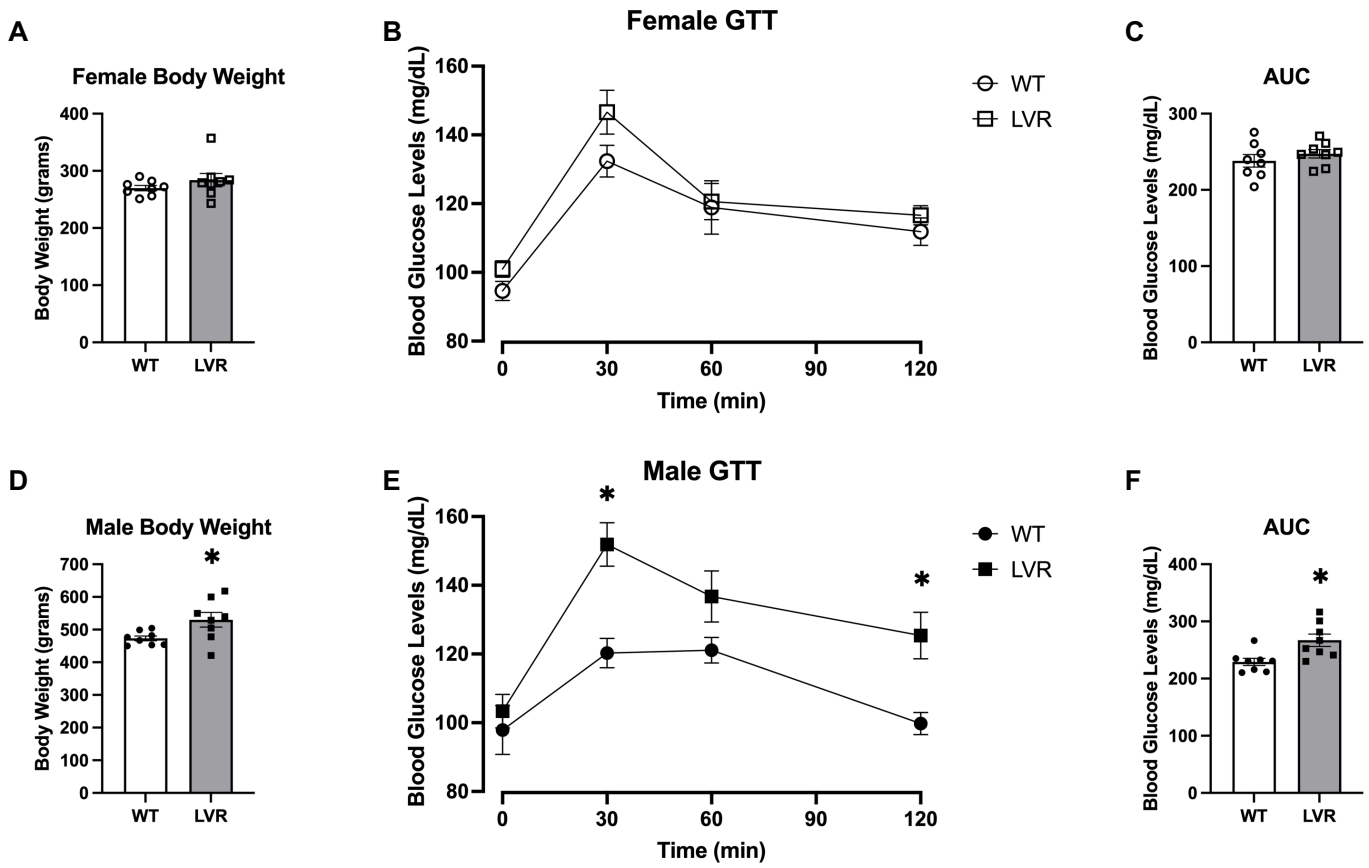
Male LVR display deficits in glucose uptake and increased bodyweight. **(A)** Bodyweight of 4-month-old female WT and LVR (*N* = 8). **(B)** Glucose tolerance testing results in female WT and LVR. **(C)** Area under the curve analysis of glucose tolerance testing in females. **(D)** Bodyweight in male WT and LVR (*N* = 8) evaluated by Student’s *t*-test. **(E)** Glucose tolerance testing results in male WT and LVR evaluated by RM 2-way ANOVA. **(F)** Area under the curve analysis of glucose tolerance testing in males evaluated by Student’s *t*-test. ^*^Denotes significant difference between groups. Values expressed as mean + SEM.

Two of the primary systemic risk factors for AD are insulin resistance and obesity (Armstrong, 2019; Edwards et al., 2019). Therefore, this portion of experiments focused on evaluating insulin sensitivity, glucose uptake, and body weight in 16-week-old male and female LVR and WT rats. Regarding the female LVR rats, they did not show a significant difference relative to WT rats when undergoing a Glucose Tolerance Test (GTT) although there was a trending main effect for a group difference based on a RM 2-way ANOVA [*F*(1, 55) = 3.88; *p* = 0.054] (Figure 6B). There were also no differences in Area under the Curve analysis of the GTT (*p* = 0.364) (Figure 6C) or bodyweight between LVR and WT (*p* = 0.284) (Figure 6A). In contrast, Male LVR displayed impairments in glucose uptake based on a GTT with a significant main effect for group [*F*(1, 14) = 12.75; *p* = 0.0031] and trending interaction [*F*(3,41) = 2.632; *p* = 0.063] (Figure 6E). Area Under the Curve analysis corroborated these findings by showing male LVR have significantly higher blood glucose levels in response to a glucose bolus relative to WT (*p* = 0.009) (Figure 6F). Male LVR also had significantly higher bodyweights relative to age-matched WT (*p* = 0.031) (Figure 6D). This indicates that male LVR show signs of insulin resistance and obesity likely increasing their risk for AD while female LVR, relative to same-sex controls, were relatively unaffected potentially due to the protective effects of estrogen in the periphery (de Paoli et al., 2021). We see very distinct effects of selecting for physical inactivity between male and female LVR in which female LVR primarily display significant deficits in brain health; whereas male LVR display systemic issues like insulin resistance and obesity yet are relatively spared from cognitive deficits.

## 4. Discussion

Human meta-analyses have demonstrated that physical inactivity increases the risk for AD (Buchman et al., 2012; Kerr and Booth, 2022). However, this is one of the first rodent studies to characterize the physiological changes associated with the genetic enrichment of physical inactivity related genes. Herein, we encompass how selective breeding for a physical inactivity phenotype interfaces with neurodegenerative disease risk across several important levels ranging from behavioral to cellular and molecular changes that occur within the hippocampus, providing strong evidence for a genetic link between physical inactivity and AD risk. These findings are highly relevant as physical inactivity levels continue to climb in parallel fashion with the prevalence of AD; we hypothesize that physical inactivity levels are a primary contributor to the increase in AD prevalence (Alzheimer’s Statistics, 2019; Physical Activity, 2020). Importantly, these studies were performed on sedentary rats suggesting that any changes we see are due to heritable genetic or epigenetic changes associated with the selection of high and low physical activity phenotypes.

We provide evidence that female LVR show deficits in learning and memory, and reductions in hippocampal neurogenesis, mitochondrial respiration, and synaptic LTP that are likely impacted by reduced GluA1 expression levels. Surprisingly, selecting for low physical activity almost exclusively affected the brains of female LVR as the male LVR appear to be spared from most cognitive deficits relative to within sex controls, despite the presence of systemic risk factors like insulin resistance and obesity. This replicates what is seen in humans as women are far more susceptible to AD (Alzheimer’s Association, 2020); our unique LVR model may provide a novel way of teasing apart these sex differences in neurodegenerative disease risk. On the other hand, we contribute novel evidence to support that selecting for high physical activity preference produces a heritable neuroprotective effect on the brain by enhancing insulin signaling and glucose metabolism in female HVR. Together, our work suggests the presence of a strong link between the genes associated with physical activity and brain health, which could potentially play a significant role in moderating the aging brains susceptibility to neurodegenerative diseases.

### 4.1. Female LVR have deficits in learning and memory, while HVR shown signs of enhancement

The consistently longer latencies and errors made across trials when attempting to locate the escape box coupled with the inability of LVR to recognize the novel object builds on similar findings by both Wikgren and Mäkinen et al. These groups used Britton and Koch’s Low-Capacity Runner (LCR) and High-Capacity Runner (HCR) rat models and demonstrated that LCR had deficits in cognitive flexibility relative to HCR, supporting our findings (Wikgren et al., 2012; Mäkinen et al., 2023). Interestingly, the latter study saw deficits in both male and female LCR relative to HCR in pre-pulse inhibition and contextual fear conditioning memory tests. However, one explanation for this discrepancy in findings could be due to the different method of breeding selection, as LCR and HCR are bred for aerobic capacity whereas LVR and HVR in the present study are bred for desire to voluntarily run. In the study mentioned above, LCR and HCR with running wheel access run similar distances, emphasizing the stark contrast between these selective breeding lines as female LVR run roughly 1% of the distance that HVR do when given running wheel access. In our study, we find that selecting for physical activity preference has the greatest effect on female offspring, as male LVR and HVR performed relatively similar to WT in Barnes maze testing. This is not entirely surprising as the running phenotype is more disparate between female LVR and HVR than male LVR and HVR, further supporting that the differences in learning and memory seen in these experiments are driven by running phenotype. On the other hand, our female HVR performed as well as, or better than, WT in learning and memory tasks. To the best of our knowledge, we are the first to show that selecting for high and low physical activity produces robust divergent changes in learning and memory that are not only different from one another, but that of their WT counterparts as well. To further analyze the benefits of selecting for high physical activity status, future studies should utilize aged HVR and WT as we hypothesize the cognitive differences will become increasingly apparent in an aging model.

### 4.2. Female LVR have deficits in hippocampal neurogenesis and mitochondrial respiration

As stated previously, mitochondrial dysfunction and reductions in LTP and adult neurogenesis in the hippocampus are both key signs of AD (Swerdlow et al., 2010; Mu and Gage, 2011). Here, we found that female LVR rats had reductions in the number of immature neurons present in the dentate gyrus, reductions in state 2, state 3 – complex I, and uncoupled respiration, as well as deficits in LTP induction. This builds on previous transcriptomics findings by our lab showing that LVR have reductions in gene networks involved in proliferation and neurotransmission in the hippocampus relative to WT (Kelty et al., 2021). Choi et al. (2014) made similar findings in 25-month-old male LCR rats whereby they had significantly fewer hippocampal neurons present, and also showed deficits in learning and memory (evaluated by Y maze spontaneous alternation) and found reductions in complex III and state 3 respiration in the hippocampus relative to HCR rats. Interestingly, we only saw differences between female LVR and WT, this discrepancy is potentially due to the younger age of the animals in our experiments. We also had the surprising finding that hippocampal mitochondrial respiration in female WT was greater than that of male WT. A couple studies support these findings, Gaignard et al. showed that progesterone acts to enhance mitochondrial respiration in the female brain; further, they went on to show that females have higher whole brain mitochondrial respiration and lower oxidative stress (Gaignard et al., 2018). Guevara et al. (2009) found that while aged female rats had less mitochondrial content compared to male rats, they had greater mitochondrial complex activity, and their mitochondria were more resistant to oxidative damage. These findings emphasize the important role of female sex hormones in neuroprotection. An important area of study will be delineating the function and health of brain mitochondria with and without the presence of female sex hormones as this could provide a greater understanding on the increased rate of AD in post-menopausal women (Mosconi and Brinton, 2018; Scheyer et al., 2018). To the best of our knowledge, our study is the first to report that mitochondrial respiration in the hippocampus differs between male and female rats, whether or not this could play a role in the sex differences seen in AD prevalence and progression is currently unclear and deserves further exploration. Overall, our findings show that female LVR display reductions in hippocampal neurogenesis, LTP induction, and mitochondrial respiration, all hallmark signs of AD.

### 4.3. AMPA receptor GluA1 expression is reduced in the dentate gyrus of female LVR

The above findings led us to evaluate the molecular changes in the dentate gyrus of male and female LVR and HVR rats as it relates to the signs of AD. The reduction in GluA1 protein expression in female LVR stands out as a molecule of interest as recent work has implicated AMPA receptor dysregulation to be one of the first steps in AD pathogenesis (Guntupalli et al., 2016; Qu et al., 2021). The reduced GluA1 levels at the protein level coupled with deficits in LTP induction makes GluA1 likely one of the key molecular drivers for the learning and memory deficits displayed by female LVR, as AMPA receptors are essential for the initial depolarization of the cell for LTP to occur and for synaptic plasticity in general (Sprengel, 2006; Antunes and Simoes-De-Souza, 2018). The fact that we see an elevated expression profile of GluA1 in female HVR at the transcript level suggests that GluA1 expression is partially dependent upon the selection for high or low physical activity preference, meaning that GluA1 expression in the dentate gyrus appears to have a heritable genetic or epigenetic connection with physical activity. Based on these differences, we hypothesize that AMPA receptor signaling likely plays a key role in producing the two distinct phenotypes we see between our LVR and HVR lines.

The absence of differences between LVR and WT in APP, Tau, and pTau expression is not too surprising based on the age of the experimental animals, which was chosen for these experiments as it was conducive to behavioral testing performance and compliance. What is interesting, however, is that these findings provide support for the mitochondrial cascade hypothesis by demonstrating that in our LVR model, which display signs of neurodegeneration and cognitive impairment, brain mitochondrial dysfunction is present absent of, or before, any changes in APP processing or Tau phosphorylation have occurred (Swerdlow et al., 2010). Future work will focus on aging LVR, WT, and HVR to determine if amyloid beta and Tau hyperphosphorylation develops at different rates between these selective breeding lines and will provide insight as to whether or not elevated Tau levels in HVR are pathological or physiological.

### 4.4. Enhancements in brain insulin signaling and glucose metabolism in female HVR

While the role of brain insulin resistance is still poorly understood as it relates to AD, insulin signaling in the brain has been demonstrated to be an important factor in the pathogenesis of AD (Neumann et al., 2008). In fact, studies have shown intranasal administration of insulin improves memory in both animal models and in human clinical trials (Chapman et al., 2018; Gabbouj et al., 2019). Brain glucose metabolism has also been shown to be severely dysregulated in AD, likely due to oxidative damage (Butterfield and Halliwell, 2019). To the best of our knowledge, we are the first to show that selective breeding for high physical activity produces enhancements in the expression of transcripts involved in brain insulin signaling and glucose metabolism. The lack of differences noted in our male HVR for these outcomes was surprising, but we hypothesize that this could be due to female rats being more physically active than male rats on average. Our female HVR rats run ~37% further than their male counterparts based on generation 32 data. This could help explain why many of the differences seen in this study are most apparent in females due to their more distinct running phenotype relative to males. We hypothesize that these enhancements, along with increases in hippocampal wet weight, produce a resiliency against AD in our female HVR.

### 4.5. Signs of systemic insulin resistance and obesity in male LVR

Male LVR may have remained relatively unscathed across most of our testing, however, what we see going on in the periphery tells a different story. As mentioned earlier, Type II Diabetes, insulin resistance, and obesity are all major risk factors for AD development (Tumminia et al., 2018; Armstrong, 2019; Edwards et al., 2019). LVR males demonstrate signs of all of the above with poor glucose uptake following administration of a glucose bolus as well as elevated bodyweights relative to WT. Future studies should analyze similar outcomes in aged male LVR as these issues in the periphery likely lead to cognitive deficits at a later age. The dichotomy that exists between the male and female LVR is an interesting one worth further study, as it seems female LVR are protected from issues in the periphery while males are protected from issues in the brain. This provides an excellent model for teasing apart sex differences across various risk factors for AD.

Overall, the work presented here provides evidence that selective breeding for high and low physical activity produces enhancements and deficits on cognition, respectively, and likely mediates neurodegenerative disease risk. We propose that LVR represent a unique model of AD-like neurodegeneration that could be used to study the relationship between various risk factors associated with physical inactivity, sex differences, and neurodegeneration. Future work should also focus on elucidating the genetic or epigenetic links that are present between the physical activity levels of previous generations and subsequent brain health. These findings should also serve as a demonstration of the importance of maintaining proper physical activity levels, as our work suggests the harms of physical inactivity stretch even beyond the culprit.

## Data availability statement

The raw data supporting the conclusions of this article will be made available by the authors, without undue reservation.

## Ethics statement

The animal study was reviewed and approved by the University of Missouri Animal Care and Use Committee.

## Author contributions

NK: conception and study design, experimentation, analysis, writing, and editing. TK, XM, and TC: experimentation and editing. DK and FB: funding, resources, analysis, and editing. RR: funding, resources, and editing. All authors contributed to the article and approved the submitted version.

## Funding

FB was supported in part with resources and the use of facilities at the Harry S. Truman Memorial Veterans Hospital in Columbia, Missouri. Resources for immunohistochemistry and electrophysiology experiments were supported by grants NIH NHLBI HL128454 and HL098602 (DK).

## Conflict of interest

The authors declare that the research was conducted in the absence of any commercial or financial relationships that could be construed as a potential conflict of interest.

## Publisher’s note

All claims expressed in this article are solely those of the authors and do not necessarily represent those of their affiliated organizations, or those of the publisher, the editors and the reviewers. Any product that may be evaluated in this article, or claim that may be made by its manufacturer, is not guaranteed or endorsed by the publisher.
